# *Plasmodium yoelii* iron transporter PyDMT1 interacts with host ferritin and is required in full activity for malarial pathogenesis

**DOI:** 10.1186/s12915-023-01776-y

**Published:** 2023-12-05

**Authors:** Mengjiao Zhong, Bing Zhou

**Affiliations:** 1 School of Life Sciences, Tsinghua University, Beijing, 100084 China; 2 Faculty of Synthetic Biology, Shenzhen Institute of Advanced Technology, Chinese Academy of Sciences, Shenzhen, 518055 China

**Keywords:** *Plasmodium yoelli*, Iron, PyDMT1, Ferritin, Artemisinin

## Abstract

**Background:**

The rapid reproduction of malaria parasites requires proper iron uptake. However, the process of iron absorption by parasites is rarely studied. Divalent metal transporter (DMT1) is a critical iron transporter responsible for uptaking iron. A homolog of human DMT1 exists in the malaria parasite genome, which in *Plasmodium yoelii* is hereafter named PyDMT1.

**Results:**

PyDMT1 knockout appears to be lethal. Surprisingly, despite dwelling in an iron-rich environment, the parasite cannot afford to lose even partial expression of PyDMT1; PyDMT1 hypomorphs were associated with severe growth defects and quick loss of pathogenicity. Iron supplementation could completely suppress the defect of the PyDMT1 hypomorph during in vitro culturing. Genetic manipulation through host ferritin (Fth1) knockout to increase intracellular iron levels enforced significant growth inhibition in vivo on the normal parasites but not the mutant. In vitro culturing with isolated ferritin knockout mouse erythrocytes completely rescued PyDMT1-hypomorph parasites.

**Conclusion:**

A critical iron requirement of malaria parasites at the blood stage as mediated by this newly identified iron importer PyDMT1, and the iron homeostasis in malarial parasites is finely tuned. Tipping the iron balance between the parasite and host will efficiently kill the pathogenicity of the parasite. Lastly, PyDMT1 hypomorph parasites were less sensitive to the action of artemisinin.

**Supplementary Information:**

The online version contains supplementary material available at 10.1186/s12915-023-01776-y.

## Backgroud

Malaria is a life-threatening infectious disease. Prevention and treatment of malaria is still a significant health issue of human concern. *Plasmodium* is the pathogen of malaria, and research on its metabolic process will benefit our understanding of this deadly organism and provide new insights into new antimalarial drug development.

Iron is an essential trace element for almost all organisms, including malaria parasites, which require iron for growth and development. It was once believed that after hemoglobin degradation, heme produced in the food vacuole is absorbed and utilized by the malaria parasite for iron [1, 2]. Heme metabolism to ferrous iron and biliverdin requires HO (heme oxygenase) [3]. However, although the genome contains a gene encoding an HO-homologous protein, the amount of biliverdin produced by heme degradation in malaria parasite is extremely low [4]. Expression of PfHO in *E. coli* revealed that it binds heme, but the process of degrading heme does not occur, suggesting that malaria parasites could not use heme iron. The absorption and utilization of non-heme iron may thus have been taken into account by malaria parasites. ZIPCO, a malarial metal transporter belonging to the ZIP (zinc-regulated, iron-regulated transporter-like proteins) family, is the first iron importer found in malaria parasites. However, ZIPCO seems to be only required in the liver stage, as deletion of ZIPCO resulted in no apparent blood stage defects [5]. PbVIT, homolog of CCC1 in yeast, acts as a detoxifier to transport iron out of cytosol in malaria parasites [6]. PfCRT (the chloroquine resistance transporter) protein expressed in Xenopus oocytes has been shown to be capable of transporting iron ions [7]. However, PfCRT mutation is known to be resistant to chloroquine by transporting chloroquine from the food vacuole to the cytosol, reducing the effect of chloroquine [8]. This, nevertheless, has not been shown to be related to iron in vivo. We hypothesize that either the malaria parasites may express a particular iron importer that is important for iron absorption and utilization at the blood stage, or, less likely, no iron transporter is required at all at this stage.

During the asexual stage, malaria parasites infect hepatocytes or red blood cells and uptake iron from the host for their growth and amplification. Changes in the host iron status will affect the iron availability for the parasites [9–11]. Studies on murine malaria models have uncovered that secondary liver infections are unlikely to happen even when a tiny amount of malaria parasites exists in the blood [12]. Besides immunity, another reason is believed to be that parasites in the blood induce the host to secrete hepcidin, leading to a reduction in iron absorption, and a decrease of iron content in the hepatocyte blocks parasites’ reinfection in the liver. Notably, deletion of ferroportin to increase iron levels within erythrocytes promoted parasite growth in the blood stage [13]. These findings suggest that the host and malaria parasites have a strong interaction in competing for iron. The host expresses a set of iron-related proteins to regulate intracellular iron levels. Under the invasion of malaria parasites, the host may adjust the expression of these proteins trying to limit iron to the intracellular parasite.

Divalent metal transporter (DMT1) is a critical iron transporter with 12 transmembrane structures responsible for iron uptake in many organisms [14–16]. Through bioinformatics analysis, we found a homolog of human Dmt1 in the malaria parasite genome, which we named PyDMT1 in *P. yoelii*. To test whether PyDMT1 acts as an iron importer in *P. yoelii*, we managed to generate a partial loss of function allele of PyDMT1 by a single crossover to the regulatory region of *Pydmt1*. This mutation caused a partial loss of PyDMT1 expression but resulted in severe defects, rescuable by the iron supplement. Moreover, inducible deletion of ferritin in mice could also elevate the free iron within reticulocytes and revert the growth defect of the *Pydmt1* mutant parasites. Notably, the *Pydmt1* mutant parasites had altered sensitivity towards artemisinins.

## Results

### Plasmodium genomes contain a DMT1 homolog, which resides on the vacuole and can functionally complement yeast smf3 for its iron-deficient phenotype

**Fig. 1:**
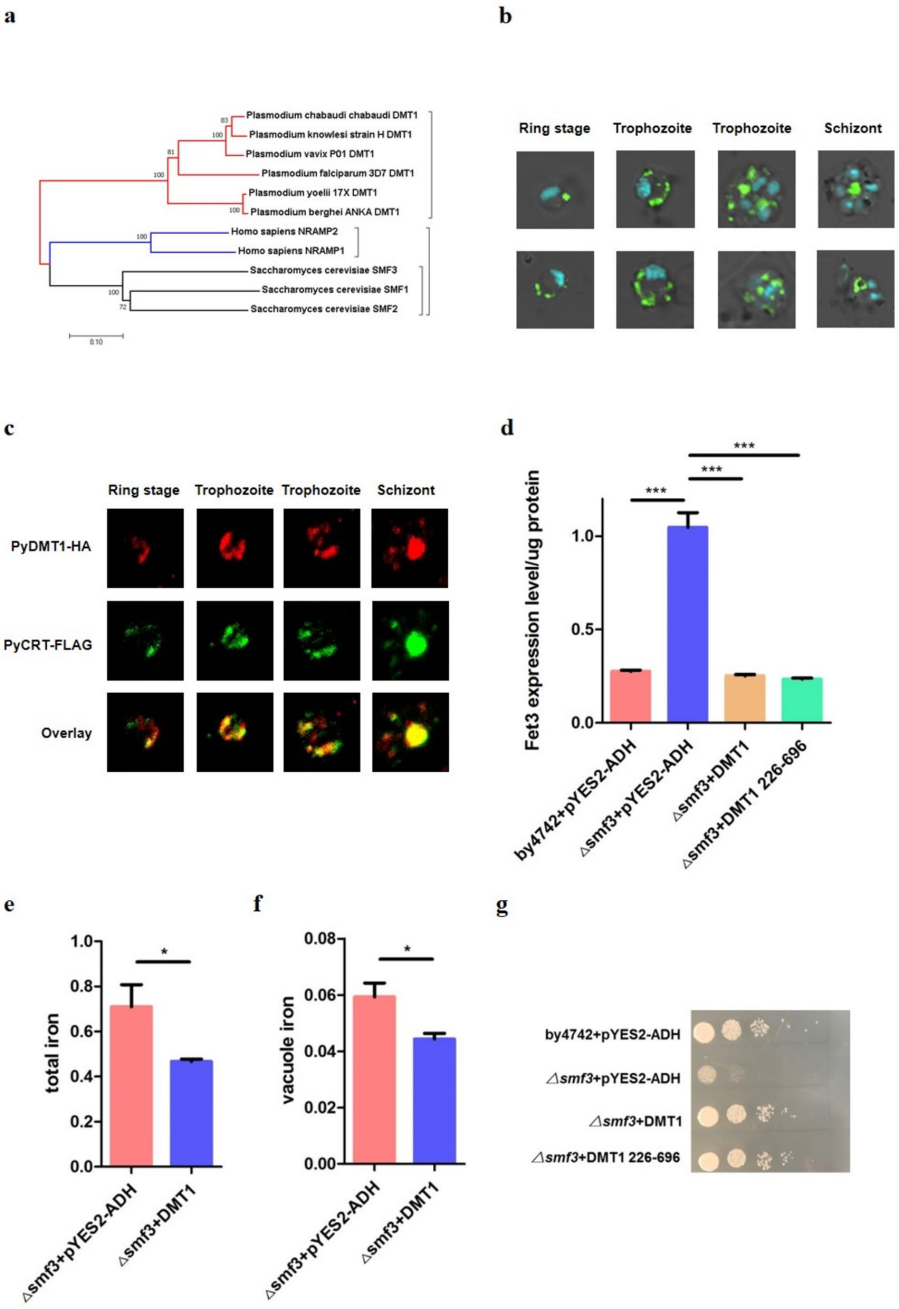
PyDMT1 resides in the vacuole and could functionally rescue the iron defect of △*smf3* yeast. **a** Phylogenetic analyses of *Plasmodium* DMT1 proteins with human and yeast (*S. cerevisiae*) homologs. The phylogenetic tree was generated with MEGA (Molecular Evolutionary Genetics Analysis). All *Plasmodium* species examined contain one DMT1 homolog. **b** Intraparasitic PyDMT1 localizes to Hz-containing DVs in *P. yoelii*. Shown here are a merge of DIC, Hoechst 33342 nuclear stain (blue), and the signal of tagged PyDMT1(green). **c** Indirect immunofluorescence assays of *PyDMT1-HA* and *PyCRT-FLAG* in the blood stage. The endogenous PyDMT1 gene fused to HA and the food vacuole marker *PyCRT-FLAG* were imaged. Shown are the HA channel (red, first row), the FLAG channel (green, second row), and a merge of both signals (third row). **d** Complementation of △*smf3* yeast iron deficiency with PyDMT1 and an N-terminal truncated PyDMT1. The regulatory region of *FET3* (− 300 bp–6 bp) directed LacZ activity (to indicate *FET3* expression response). △*smf3* and the parent strain by4742 transformed with the empty vector pYES2-ADH were the controls. Shown are mean values ± SD. ***P < 0.001; one-way ANOVA and Tukey’s multiple comparison test. *N* = 3. **e** Whole-cell iron content in △*smf3* yeast expressing PyDMT1. Shown are mean values ± SD. *P < 0.05; *t*-test. *N* = 3. **f** Iron content of the vacuole from △*smf3* yeast expressing PyDMT1yeast. Shown are mean values ± SD. *P < 0.05; one-way ANOVA *t*-test. *N* = 3. **g** Growth complementation of △*smf3* yeast with the expression of PyDMT1 or an N-terminal truncated PyDMT1

Bioinformatic analysis shows that all *Plasmodium* genomes encode one gene product with homology to *Saccharomyces cerevisiae* (the yeast) Smf1-3, *Homo sapiens* Nramp1 and Dmt1 (Nramp2) (Fig. 1a). This Dmt1 homolog is PF3D7_0523800 in *P. falciparum* and PY17X_124180 in *P. yoelii* (PyDMT1). PyDMT1 shares 29.53% amino-acid sequence identity with *Homo sapiens* Dmt1, and 24.94% with *S. cerevisiae* Smf3. PyDMT1 is predicted to have twelve transmembrane domains. In order to reveal the expression and subcellular localization of PyDMT1 protein, we fused a HA tag in frame with the endogenous PyDMT1 coding region at the carboxy terminus by CRISPR-Cas9 [17, 18] in *P. yoelii* 17X, and obtained a recombinant PyDMT1-HA *P. yoelii* strain (Fig S1a-b). Confocal analysis after immunostaining with HA antibody showed that PyDMT-HA surrounded the hemozoin in the blood stage (Fig. 1b), implying that PyDMT1 is likely localized on the membrane of the food vacuole. The transcription level of DMT1 in the PyDMT1-HA parasites was consistent with that of the wild-type parasites; there was no significant difference in growth between the two strains after infecting mice in the blood stage (Fig S1e-f), which means that the HA fusion has unlikely disrupted the function of PyDMT1.

*Plasmodium* CRT (chloroquine-resistant transport) is known to localize on the vacuolar membrane, responsible for chloroquine resistance [19]. To further confirm that PyDMT1 is localized on the food vacuole membrane, we fused a FLAG tag at the C-terminal of CRT by single crossover to label the food vacuole (Fig S1c-d). Confocal imaging revealed that PyDMT1 indeed mainly colocalized with CRT at all blood stages (Fig. 1c), and slightly with the Golgi marker Rab6 (Fig S1h), but completely not with the endoplasmic reticulum marker PMV (Fig S1g). During the schizont stage, a small amount of PyDMT1 signal is found in the merozoites, mainly in the hemozoin region. These data strongly suggest that PyDMT1 mainly localizes on the digestive vacuole membrane.

Sequence comparison indicates that *S. cerevisiae* Smf3 bears a closer resemblance to PyDMT1 than Smf1 and Smf2 (Fig. 1a), consistent with the finding that Smf3 is located on the vacuole. In the yeast, Smf3 transports iron from the vacuole into the cytosol, and Smf3 deletion makes yeast iron deficient, accompanied by elevated Fet3 expression [20]. The expression of Fet3 can thus act as a sensitive indicator for iron deficiency. To assess whether PyDMT1 could rescue the iron deficiency phenotype in △*smf3*, a yeast expression vector (pYES2-ADH) with a codon-optimized full-length *Pydmt1*(696 aa) and a 5’-truncated *Pydmt1*(470 aa) containing all 12 transmembrane domains were transformed into △*smf3* yeast*.* A parallel transformation with the empty vector was used as the negative control. *FET3* promoter activity was monitored by a reporter construct wherein the *FET3* gene promoter was fused to *LacZ* (β-galactosidase gene Z). The results showed that both the full-length *Pydmt1* and 5’-truncated *Pydmt1* conferred significant rescue to the △*smf3* (Fig. 1d). To confirm that expressing PyDMT1 in *△smf3* yeast enables iron transport from the vacuole to the cytosol, we quantified the whole-cell iron and vacuole iron contents. Consistently, the whole cell and vacuole iron levels were both lower when expressing a codon-optimized PyDMT1 (Fig. 1e, f). Plate growth assays also showed that expressing PyDMT1 restored the growth of the △smf3 strain (Fig. 1g), indicating that PyDMT1 can function heterogeneously in *S. cerevisiae* and act as an iron importer there.

### PyDMT1 is critical for parasite development in the blood stage

**Fig. 2:**
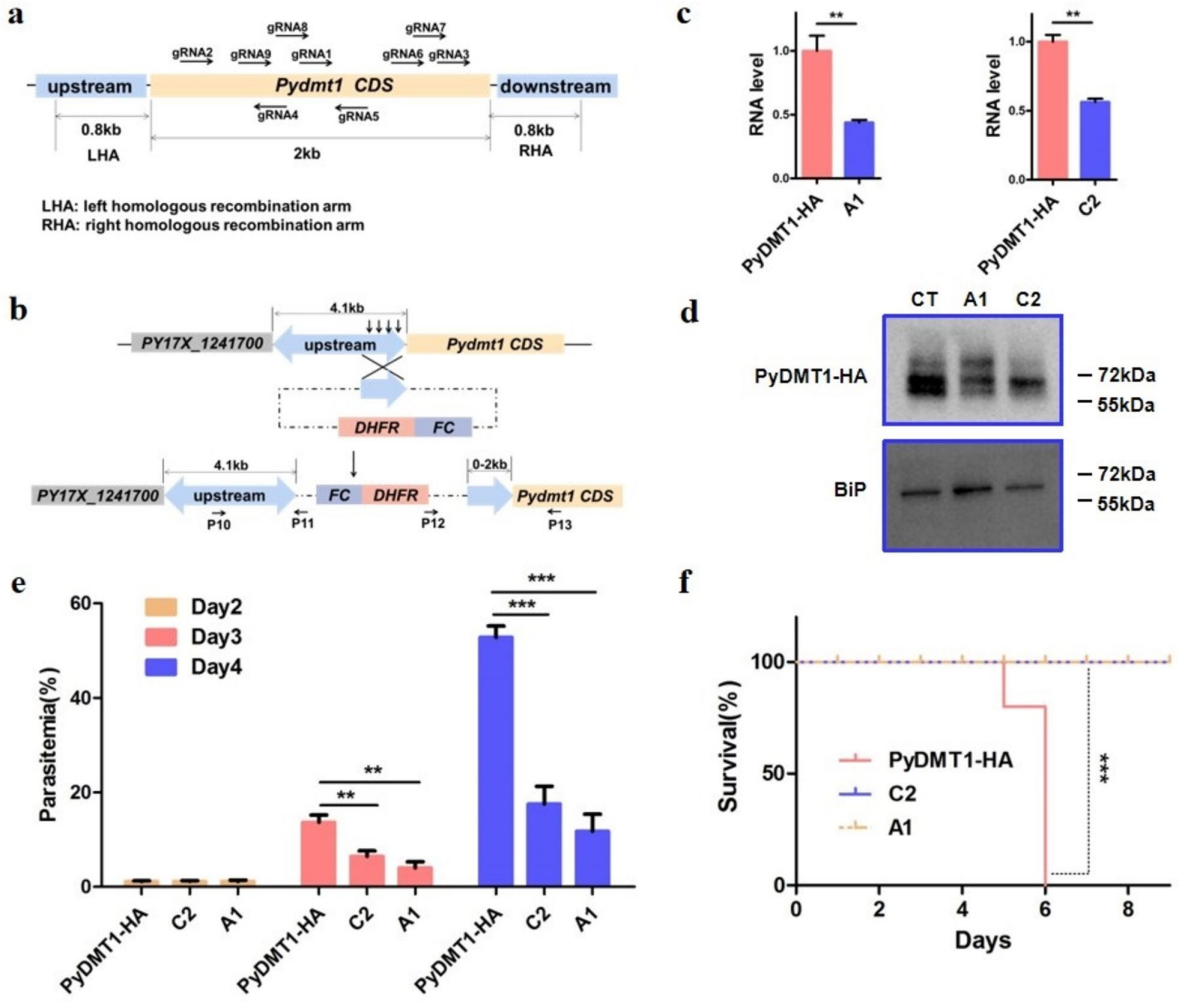
PyDMT1 hypomorphs are associated with reduced growth and suppressed pathogenicity. **a** A schematic showing positions of nine guide RNAs and homologous recombination arms designed for *Pydmt1* knockout. Each guide RNA and flanking homologous recombination arms were cloned in the vector expressing Cas9, and parasites were selected with pyrimethamine after transfection. All failed in repeated trials. Different homologous recombination arms were also tried in vain. **b** Single crossover strategy for PyDMT1 knockdown in *P. yoelii*. The strategy was designed in such a way that after a successful crossing-over, the 4.1-kb region upstream from the translation start of the neighboring gene would remain intact, leaving only the *Pydmt1* regulatory region truncated at the insertion point. Arrows indicate various truncations upstream of *Pydmt1* CDS had been tried to make the hypomorphic mutant. **c** qPCR detection of *Pydmt1* transcript levels for PyDMT1 hypomorphic clones A1 and C2. The control parasites of *PyDMT1-HA* and hypomorphic clones A1and C2 were purified from mouse blood and subjected to qPCR using primers targeting the 3’ segment of PyDMT1. PyDMT1 transcript abundance was normalized to tubulin. Shown are mean values + / − SD. **P < 0.01; *N* = 3. **d** Protein levels of *Pydmt1*-A1 and *Pydmt1*-C2 as detected by western blots. HA antibody was used for detection. The specificity of the HA antibody was demonstrated with none-HA carrying parasite strain (Figure S2d), suggesting all the three bands from the western blot were derived from PyDMT1. **e** Reduced growth of the *Pydmt1* hypomorphic mutants*.* Parasitemia of BALB/c mice following infection (i.v.) with 10^6^ PyDMT1 or PyDMT1 hypomorphic iRBC was determined by counting iRBC in Giemsa-stained blood smears(*N* = 5, one-way ANOVA and Tukey’s multiple comparison test, **P < 0.01, ***P < 0.001). **f** Loss of pathogenicity for the *Pydmt1* hypomorphic mutants. Shown here is the survival of BALB/c mice inoculated with 10.^6^ control *P. yoelii* or hypomorphic PyDMT1 parasite-infected iRBC (*N* = 5, log-rank test, ***P < 0.001)

To clarify the physiological function of PyDMT1 in *Plasmodium*, we attempted to knock out PyDMT1 in *P. yoelii*17X by CRISPR-Cas9 [17, 18]. Nine guide RNAs were designed to cover the *Pydmt1* gene (Fig. 2a, Table S1), and each guide RNA and homologous recombination arms were independently electro-transfected five times. Unfortunately, no *Pydmt1* knockout parasites were obtained in all these trials. We, therefore, suspect that PyDMT1 may be indispensable for the viability of parasites.

For an essential gene, the alternative strategy is to generate a partial loss of function mutant (hypomorph). Truncating the regulatory region by inserting a selectable cassette into the upstream of a gene could reduce its expression [21]. We decided to insert an *hdhfr*(coding for the human dihydrofolate reductase) selectable cassette at the *Pydmt1* locus upstream of the coding region of *Pydmt1-HA* to knock down its expression. In the *Plasmodium* database, we found that *Pydmt1* shares the same regulatory region of approximately 4.1 kb with an adjacent gene, PY17X_1241700, whose function remains unknown. To minimize the undesirable effect on this neighbor gene, we designed a single insertion strategy to shorten the regulatory region of *Pydmt1* without altering the entire 4.1 kb regulatory region of PY17X_1241700 (Fig. 2b). Multiple attempts to put the cassette in at − 400, − 600, − 800, − 1200, and − 1600 bp upstream of *Pydmt1* were unsuccessful. Nevertheless, we finally obtained a recombinant clone by inserting the *hdhfr* cassette at the − 2000 bp position (Fig. 2b). After limiting dilution and mice infection, we obtained six independent clones (Fig S2a-b), among which two clones (*Pydmt1*-A1 and *Pydmt1*-C2) were selected for *Pydmt1* expression analysis. Inserting *hdhfr* at the − 2000 bp led to a reduction of *Pydmt1* expression in the clones, demonstrated at both the mRNA and protein levels (Fig. 2c, d). This insertion, however, had little influence on the mRNA expression of the flanking gene PY17X_1241700 (Fig S2c). Amazingly, this roughly halving of PyDMT1 expression led to a significantly reduction of parasitemia; 3 days after infection the parasitemia for *Pydmt1*-C2 clone and *Pydmt1*-A1clone was each reduced by 50 and 60%, respectively (Fig. 2e). Furthermore, BALB/c mice infected with the hypomorphic PyDMT1 parasites survived significantly longer than mice infected with control parasites (Fig. 2f). These results suggest that full PyDMT1 activity is critical for *P. yoelii* pathogenicity, and even partial reduction of PyDMT1 expression would seriously cripple its lethality*.* An inference of this finding is that although iron is so critical for the pathogen’s survival, normal *P. yoelii* does not contain much higher iron levels than needed.

### PyDMT1 hypomorph could be completely rescued by ferrous supplementation in vitro

**Fig. 3:**
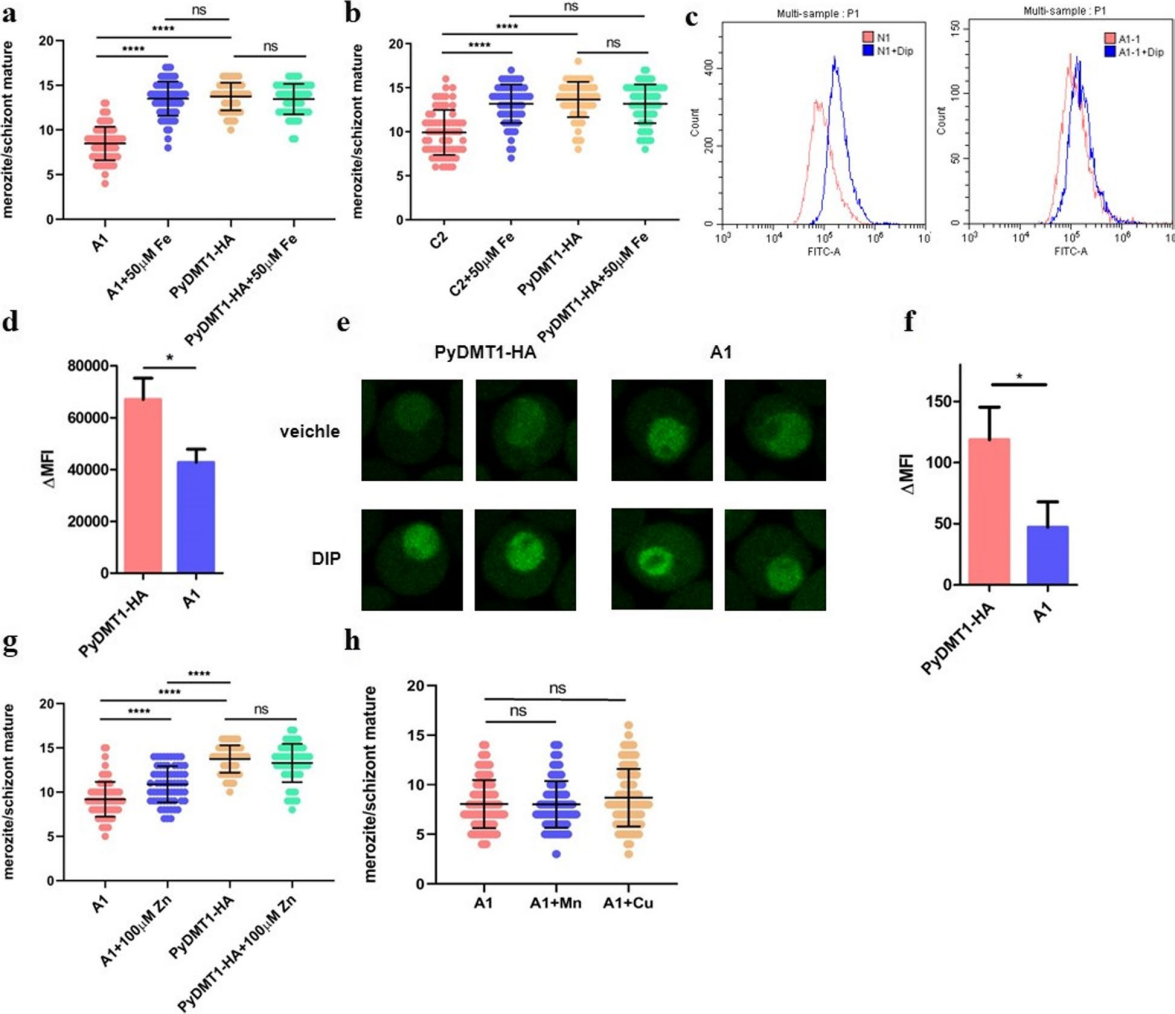
Iron could completely rescue the growth defects of PyDMT1 hypomorph in vitro. **a** Schizont maturation of *Pydmt1-*C2 rescued with 50 μM FeSO4. One hundred micromolars ascorbic acid was added to prevent oxidation/maintain Fe^2+^ status. After 18 h incubation (2 p.m. to 8 a.m., at the atmosphere of 90% N_2_, 5% O_2_, and 5% CO_2_), most parasites in early ring stage developed to mature schizont (one hemozoin is surrounded with merozoites before release). Merozoites were counted under a microscope with blood smear. Shown are values from individual parasites as well as the mean values. One-way ANOVA and Tukey’s multiple comparison test. ****P < 0.0001. *N* = 80 parasites from three independent experiments. **b** Merozoite numbers of *Pydmt1-*A1 rescued with 50 μM FeSO4 in the presence of 100 μM ascorbic acid (*N* = 80 parasites from three independent experiments, one-way ANOVA and Tukey’s multiple comparison test. ****P < 0.0001). **c** A representative histogram showing the Calcein-AM fluorescence intensities of the control *PyDMT1-HA*(N1) and the mutant *Pydmt1-*A1(A1) parasites. FITC-A in *x*-axis represents the Calcein-AM fluorescence intensity. DIP (2,2-dipyridyl) is an iron chelator and the differential ΔMFI after iron chelation is commonly used to measure the labile iron pool. **d** The LIPs of *PyDMT1-HA* and *Pydmt1*-A1 analyzed by flow cytometry, as shown in **c**. △MFI was determined by evaluating the change in mean fluorescence intensity of Calcein-AM-loaded iRBCs (Hoechst 33,342-positive subset, indicated with PB450A), after incubation with 100 μM DIP (*N* = 5, Mann Whitney test, *P < 0.05). **e** Representative images of the control PyDMT1-HA and Pydmt1-A1 parasite-infected RBCs after Calcein-AM treatment. Parasite-infected RBCs were incubated with Calcein-AM and DIP 1 h (the second row) or without (the first row). After three times washing, the iRBCs were transferred to the plate pretreated with poly-lysine, and imaged by Olympus FV3000 laser scanning confocal microscope. Quantitative data shown in **f**. **f** The LIPs of PyDMT1-HA and *Pydmt1*-A1 analyzed by confocal microscopy as shown in **e**. △MFI was determined by evaluating the change in mean fluorescence intensity of Calcein-AM in the region of parasite, after incubation with 100 μM DIP (*N* = 15 from three independent experiments, *t*-test, *P < 0.05.). **g** The schizont maturation of *Pydmt1-*A1 rescued with 100 μM Zn^2+^ (*N* = 80 parasites from three independent experiments. One-way ANOVA and Tukey’s multiple comparison test. ****P < 0.0001). **h** The schizont maturation of *Pydmt1-*A1 supplemented with 100 μM Cu^2+^ or Mn.^2+^ (*N* = 80 parasites from three independent experiments. One-way ANOVA and Tukey’s multiple comparison test. n.s. *P* > 0.05)

In mammals, DMT1 is the major iron transporter and contributes to non-heme iron uptake in most types of cell. In *Dmt1*^−*/−*^ mice, significant hepatic iron stores occurred but these mice died of anemia by day 7 [14, 16]. To test the hypothesis that the slow growth of the hypomorphic PyDMT1 parasite was due to iron deficiency caused by lower iron uptake, we counted the merozoites of the mutant *Pydmt1* parasite and the parent strain without or with exogenous iron in vitro by adding Fe^2+^. After 18 h incubation, the number of merozoites for *Pydmt1* mutant parasites (clone A1 and clone C2) was about 9, in comparison to 14 for the parent strain, consistent with the slower in vivo growth of the mutant. Meanwhile, addition of Fe^2+^ did not significantly increase the number of merozoites for the parent strain, indicating that iron was not a limiting factor for the normal parasite under our experimental condition. In contrast, iron supplements significantly increased the number of merozoites for the *Pydmt1* mutant parasite, to a comparable level with that in the control parasite (Fig. 3a, b). The data indicate that it is primarily iron deficiency and not anything else that underlies the growth defects from reduced PyDMT1 expression. Next, we compared the labile iron pool (LIP) in the control parasite and clone A1. To that end, we used a similar strategy adopted by other groups [6, 22]; iRBCs containing both the parent parasite and clone A1 were stained with the iron-sensitive fluorescent probe Calcein-AM, and the signals were analyzed by flow cytometry. The LIP of the *Pydmt1* mutant parasite iRBC was significantly lower than that of the control parasite iRBC (Fig. 3c, d, Fig S2e). Noteworthy is that Fe^2+^ quenches the fluorescence so that higher Fe^2+^ levels result in lower signals. In order to make sure that these signal changes were primarily from the parasites instead of the RBC, a confocal image was further taken. The primary signal of the Calcein-AM stain indeed appeared from the parasites (Fig. 3e, f). Furthermore, the diffuse and stronger signal across the whole parasite suggests a reduced LIP of *Pydmt1*-A1 in the cytosol.

We then determined whether the number of merozoites in *Pydmt1* mutant parasite could be reverted by other metals. Given the same nature of the different *Pydmt1* hypomorph mutants, essentially identical results obtained with A1 and C2, and the laborious amount of work involved, we chose A1 for this work. Zinc addition partially restored the number of merozoites of clone A1 (Fig. 3g), meaning that zinc supplement could, to some extent, rescue the growth defect of *Pydmt1* mutant parasites. However, adding copper or manganese ions did not affect the merozoite maturation (Fig. 3h).

Taken together, we conclude that the primary physiological function of PyDMT1 is iron uptake, and decreased PyDMT1 expression causes parasite iron deficiency, leading to severe physiological consequences. The perfect rescue of the PyDMT1 hypomorphs with iron, together with the observation of the inability of iron addition to enhance the growth of the wild-type pathogen, confirms our suspicion that the normal pathogen may obtain just enough iron for its optimal growth.

### Inducible ferritin knockout could effectively suppress the growth defect of the hypomorphic PyDMT1 parasite

**Fig. 4:**
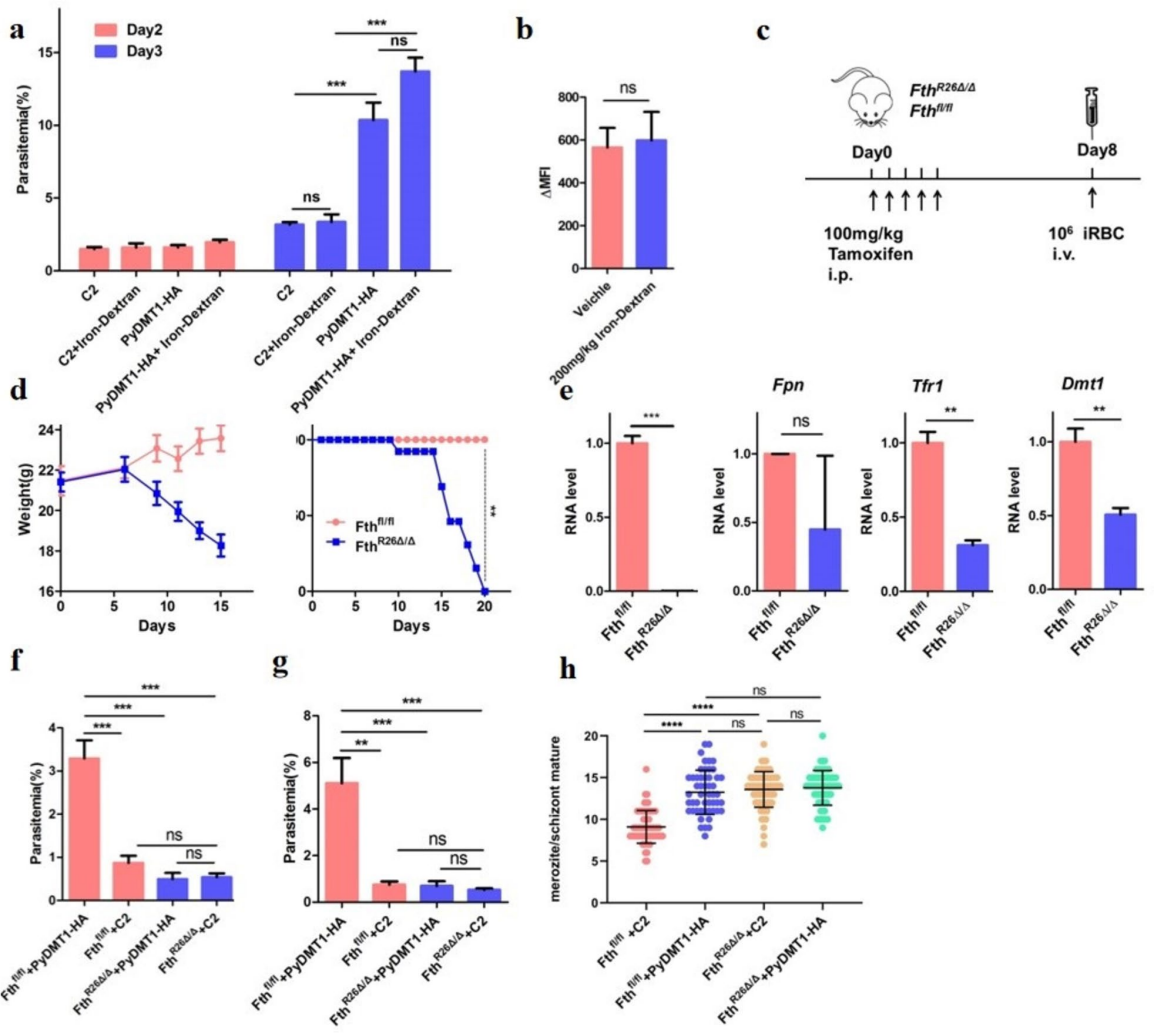
Inducible knockout of host ferritin could normalize the growth of PyDMT1 hypomorph to that of the control parasite. **a** Parasitemia of BALB/c mice infected with *PyDMT1-HA* and *Pydmt1-*C2 parasites, after injection with 200 mg/kg iron dextran. Iron dextran was injected for 3 consecutive days in advance before *PyDMT1-HA* and C2 inoculation. Parasitemia were recorded at day 2 and day 3 (the day of inoculation as day 0) (*N* = 5, one-way ANOVA and Tukey’s multiple comparison test. ***P < 0.001). **b** LIP of RBC after 5 days iron dextran injection to the mice (*N* = 3, *t*-test, n.s. no significance). **c** The schedule of ferritin (Fth1) inducible knockout. **d** Body weights and survivals (*N* = 11, log-rank test, **P < 0.01) of *Fth*^*R26△/△*^ (*N* = 14) and *Fth*^*fl/fl*^ mice, after tamoxifen administration (start at day 0). **e** Expression of *Fth1*, *Fpn*1, *Dmt1*, and *Tfr1* mRNA of blood cells in *Fth*^*fl/fl*^ (*N* = 3) and *Fth*^*R26△/△*^ (*N* = 3), 10 days after tamoxifen administration (*t*-test, ***P < 0.001, **P < 0.01), suggesting an elevation of intracellular iron in blood cells. **f** Parasitemia of *Fth*^*R26△/△*^ (*N* = 7) and *Fth*^*fl/fl*^ (*N* = 5) mice after 2 days of infection with *PyDMT1-HA* and *Pydmt1-*C2 parasites (one-way ANOVA and Tukey’s multiple comparison test, ***P < 0.001, **P < 0.01). **g** Parasitemia of *Fth*^*R26△/△*^ (*N* = 7) and *Fth*^*fl/fl*^ (*N* = 5) mice 3 days after infection with *PyDMT1-HA* or *Pydmt1-*C2 parasites (one-way ANOVA and Tukey’s multiple comparison test, ***P < 0.001, **P < 0.01). **h** Merozoite numbers of *PyDMT1-HA* and *Pydmt1-*C2 parasites cultured in *Fth*^*R26△/△*^ or *Fth*.^*fl/fl*^ blood cells (*N* = 80 parasites from three independent experiments, one-way ANOVA and Tukey’s multiple comparison test. ****P < 0.0001)

After achieving complete rescue of the *Pydmt1* mutant parasite in vitro by iron addition, we next tried this strategy in vivo. We intraperitoneally injected 100 mg/kg iron dextran into parasite-infected mice. One hundred milligrams per kilogram iron dextran injection did increase the total iron content of the plasma and erythrocytes (Fig S3a), but surprisingly had no effect on the parasitemia of the *Pydmt1* mutant parasite (Fig S3b). Concerning that 100 mg/kg iron dextran might not be sufficient to increase the intracellular iron content of the parasite, we increased the concentration of iron dextran to 200 mg/kg. Still, the parasitemia of the *Pydmt1* mutant parasite was not restored (Fig. 4a). We subsequently analyzed the LIP of the RBCs and found that it had no change after iron dextran administration (Fig. 4b), meaning elevated plasma iron levels did not translate to an increase of iron in the erythrocytes. This explains why iron dextran injection did not rescue the growth defects of our iron-deficient malaria parasites.

Ferritin, an iron storage protein, is the primary iron storage mechanism critical to iron homeostasis [23, 24]. We reasoned that the removal of ferritin would release the iron and provide an extra iron source to the cell. However, germline ferritin (Fth1) deletion results in embryonic death in mice [25]. We, therefore, tried an inducible genetic manipulation to delete Fth1 to increase intracellular iron levels. Consistent with a previous study [26], induced *Fth1* deletion by a ubiquitously expressed Rosa26-Cre (*Fth*^*R26△/△*^) resulted in rapid loss of body weight and subsequent death (Fig. 4c, d). This was not observed in the control *Fth*^*fl/fl*^ mice, which received the same inducer tamoxifen under the same schedule. Loss of ferritin repressed *Dmt1* and *Tfr1* mRNA expression of cells in the blood (Fig. 4e), consistent with the expectation of increased intracellular labile iron pool.

To test whether ferritin loss could affect the growth of our iron-deficient malaria parasites, we inoculated clone C2 and control parasite on the eighth day after tamoxifen induction and then determined parasitemia using blood smears from the second day after parasite inoculation. Interestingly, after ferritin was knocked out, the control parasite exhibited growth defects. On the fourth day, the parasitemia of the control parasite reached 5% in the control *Fth*^*fl/fl*^ mice, whereas the parasitemia in the *Fth*^*R26△/△*^ mice was less than 1%. In comparison, the parasitemias of clone C2 inoculated into the two kinds of mice were similar, but both less than 1% (Fig. 4f, g), comparable to that of the control normal parasite. It is possible that some harmful host effects after ferritin knockout were produced, which suppressed the growth of the control parasites. Why were then the mutant parasites not affected? We speculate that, on the one hand, this harmful effect may also apply to the mutant parasite, but on the other hand, the elevated free iron in the erythrocytes after ferritin knockout may have benefited the mutant growth. These two opposite actions neutralized each other for the mutant, making the difference between the control and Pydmt1 mutant parasites no longer evident.

To put this speculative thinking to the test, we cultured both the parasites with ferritin knockout erythrocytes and analyzed whether ferritin knockout erythrocytes could rescue the growth defect of *Pydmt1* mutant. Delightfully, the C2 clone growth was completely normalized after culturing with the ferritin knockout erythrocytes. Additionally, when grown with ferritin mutant erythrocytes, the control parasite’s growth deficiency in *Fth*^*R26△/△*^ likewise disappeared, demonstrating that the previously noted additional suppressive effect in vivo may indeed originate from the host independent of the erythrocytes (Fig. 4h).

### Ferritin deletion elevated the intracellular free iron level of reticulocytes

**Fig. 5:**
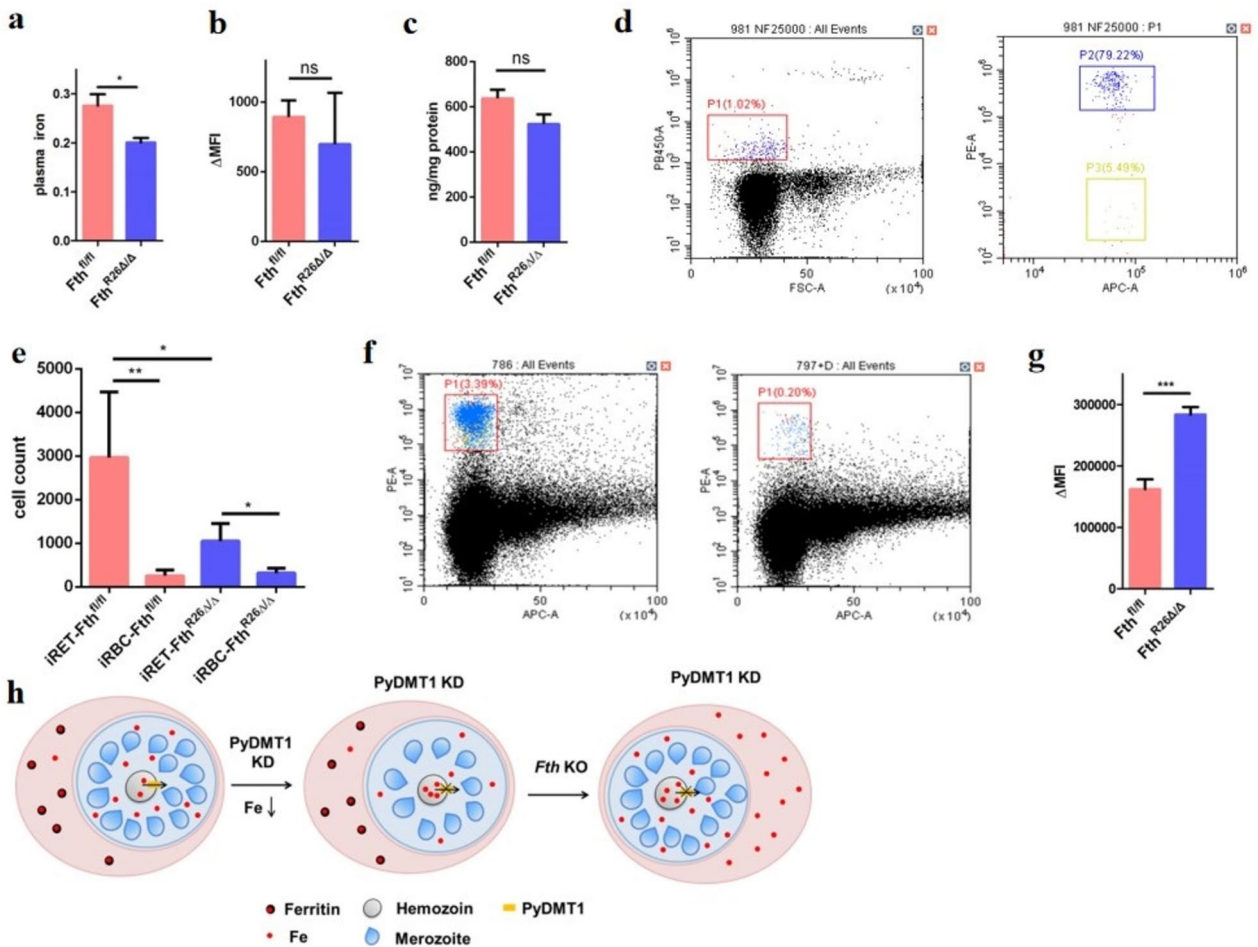
Ferritin deletion led to iron elevation in the reticulocyte and rescue of PyDMT1 hypomorph. **a** Plasma free iron of *Fth*^*fl/fl*^ (*N* = 3) and *Fth*^*R26△/△*^ mice (*N* = 3, *t*-test, *P < 0.05). **b** Free iron in the red blood cell of *Fth*^*fl/fl*^ (*N* = 4) and *Fth*^*R26△/△*^ (*N* = 4). **c** Ferritin in the red blood cell of *Fth*^*fl/fl*^ (*N* = 5) and *Fth*^*R26△/△*^ (*N* = 5). **d** Gating strategy applied to separate parasites infected reticulocytes and infected red blood cell. APC indicates TER-119-APC in *X*-axis, PE represents CD71-PE in *Y*-axis. Hoechst33342 (PB450A) to select parasites positive cell when parasitemia is up to 1%. TER-119-APC was used to label erythrocyte; CD71-PE was used to separate infected reticulocytes (iRET) and infected red blood cell (iRBC) with 1–2µL blood. **e** Cell count of iRET and iRBC (*N* = 4, *t*-test, **P < 0.01, *P < 0.05). **f** Gating strategy applied to select for reticulocytes by flow cytometry. TER-119-APC to separate erythrocyte and non-erythrocyte with 1µL blood, CD71-PE to select reticulocytes in TER-119-APC positive cell. **g** The LIP of reticulocytes from *Fth*^*fl/fl*^ (*N* = 5) and *Fth*^*R26△/△*^ mice (*N* = 5, *t*-test, ***P < 0.001). **h** A proposed model of PyDMT1 action. PyDMT1 reduction leads to iron deficiency, decreasing the number of merozoites in each schizont. Ferritin knockout elevates the intracellular iron of reticulocytes, conferring a rescue on the PyDMT1 hypomorph parasite

To confirm that after ferritin deletion the free iron was indeed elevated in parasite-infected erythrocytes, we first analyzed the LIP in the mice plasma and erythrocytes after inducible ferritin knockout. To our surprise, the plasma iron was significantly reduced (Fig. 5a), while the iron in erythrocytes did not change significantly (Fig. 5b). How could then the *Pydmt1* mutant be rescued?

After synthesizing erythroid hemoglobin, reticulocytes are formed following enucleation and remain in the bone marrow for 2–3 days before being released into the peripheral blood and then transformed into mature erythrocytes in 1–2 days. In humans, these mature red blood cells lived for about 120 days [27–29], and in mice, 38–52 days [30]. Since there is no genome in mature red blood cells, we posit that tamoxifen cannot exert any knockout effect in these cells. On the other side, mature red blood cells constitute a large percentage in the population. Therefore, as a pool, no change of free iron and ferritin in whole red blood cells may be observed only a few days after ferritin removal (Fig. 5b,c), which may only happen in the precursors of erythrocytes before their enucleation process. Moreover, 5 days are sufficient for the reticulocyte development, and we observed, as predicted, that *Plasmodium yoelii* mainly infected reticulocytes rather than the mature red blood cells (Fig. 5d, e). In other words, the iron level in the reticulocytes, which *Plasmodium yoelii* infection is susceptible to, can be increased after ferritin removal, enabling a rescue of the growth defect of *Pydmt1* mutant parasites. To test this hypothesis, we have to separate the reticulocyte minority from the mature red blood cell majority. We used an antibody to label reticulocytes to divide the blood cells into two populations (Fig. 5f). As expected, the free iron content in the reticulocytes indeed increased significantly after ferritin loss (Fig. 5g, h). This indicates a delicate balance of iron homeostasis exists between the host and pathogen.

### Pydmt1 mutant malaria parasites were less sensitive to artemisinin

**Fig. 6:**
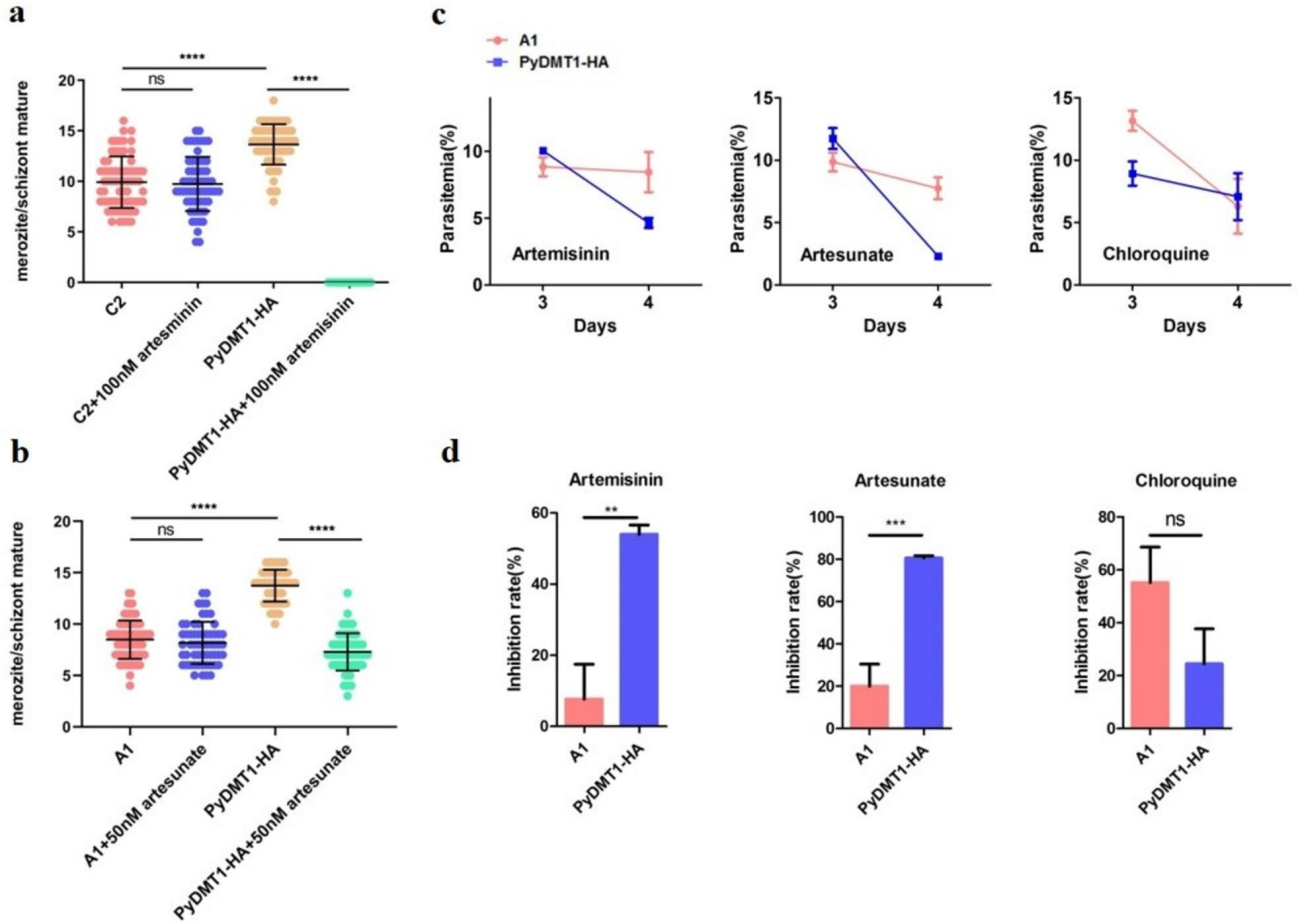
PyDMT1 hypomorph parasites were less sensitive to artemisinin. **a** Merozoite numbers of *Pydmt1-*C2 treated with 100 nM artemisinin (*N* = 80 parasites from three independent experiments, one-way ANOVA and Tukey’s multiple comparison test. ****P < 0.0001). **b** Merozoite numbers of *Pydmt1-*A1 treated with 50 nM artesunate (*N* = 80 parasites from three independent experiments, one-way ANOVA and Tukey’s multiple comparison test. ****P < 0.0001). **c** Parasitemia of BALB/c mice treated with 7.5 mg/kg artemisinin, 8 mg/kg artesunate, or 6 mg/kg chloroquine after infected with *PyDMT1-HA* and *Pydmt1-*A1 parasites. Drugs were administered when parasitemia was up to 10%. This assay is limited to just 2-day assays due to obvious immune reaction to *P. yoelii* 17X 5 days after infection*.* **d** Inhibition rate of BALB/c mice treated with 7.5 mg/kg artemisinin, 8 mg/kg artesunate, or 6 mg/kg chloroquine after infection with *PyDMT1-HA* and *Pydmt1-*A1 parasites (*N* = 5, *t*-test, ***P < 0.001, **P < 0.05)

Artemisinins are first-line antimalarial drugs. Though the precise method by which artemisinins fight malaria is still up for debate, it is widely acknowledged that the endoperoxidic bond must first be activated for the substance to have effect. Several kinds of activation mechanisms have been proposed, but essentially none contradict the involvement of iron in the process [31]. Since *Pydmt1* mutations led to serious reduction of iron in the parasites, it would be interesting to test whether these parasites are more sensitive or resistant to artemisinins. Our *Pydmt1* mutant parasites were indeed less sensitive to the action of artemisinins, both in vitro (Fig. 6a, b) and in vivo (Fig. 6c, d). Similarly, this also occurred to artesunate (Fig. 6c, d). As a negative control, the *Pydmt1* mutation posed no alterations in susceptibility to another common antimalarial drug, chloroquine (Fig. 6c, d).

## Discussion

Iron is an essential nutrient for almost all organisms. The iron absorption process by malaria parasites at the blood stage has not been reported. In this work, we found that all *Plasmodium* genomes encode a homolog of human DMT1. Expressing a codon-optimized *Pydmt1* restored iron availability in *△smf3* yeast, implying that PyDMT1 is functional in the yeast and moves iron into the cytosol. Subcellular localizations of DMT1 in mammalian cells are complex; different cells have different localizations, presumably satisfying their different physiological needs. *Plasmodium* lives within the red blood cells, primarily relying on degrading the cytosolic component of erythrocytes as the main source of nutrition. It is no wonder that PyDMT1 is positioned at the membrane of the food vacuole. This localization of PyDMT1 is consistent with the development characteristics of *Plasmodium* in the blood stage and indicates that the digestive vacuole is a critical source of iron for malaria parasites. When preparing to write the manuscript, we read an article mentioning that PfFVRT1 (PfDMT1) was indispensable in the blood stage and localized on the food vacuole [32], consistent with our results.

Malaria parasites at the blood stage dwell in an iron-rich or, more precisely, hemin-rich environment. An unexpected finding of this work is that the parasite at this stage critically depends on the absorption of the labile form of iron. PyDMT1 is indispensable for this process. Even more unexpected is that the full activity of PyDMT1 is necessary. Multiple efforts to target multiple sites of the gene failed to obtain mutant clones. Only *Pydmt1* partial loss of function or hypomorph alleles were successfully generated 2 kb upstream of the coding region, which reduced the expression of the gene by about half. This partial reduction of expression enacted much stress on the parasites, and almost eliminated their lethality. This finding suggests that in normal parasites, the expression of PyDMT1 is just enough to regulate proper amounts of iron to be imported into the cytosol. This is reasonable considering that iron excess is toxic. The interaction of ferritin and PyDMT1 also fits this scenario. The host ferritin acts to chelate or store excessive amounts of iron in the erythrocytes, and the parasite has to regulate the expression of PyDMT1 so that sufficient but not excessive iron is imported into the cytosol. This leads to the scenario that even a small tilt in balance of PyDMT1 expression results in iron insufficiency for parasite use.

The extreme vulnerability to iron deficiency from PyDMT1 suggests this might be an Achilles’ heel for malaria parasites and could work as an effective target for drug development. Our current understanding is that though multiple genes are necessary for an organism to grow, most of them are lethal only when the gene’s product is completely removed or effectively suppressed; few will display severe disability by losing half of the expression. In other words, in most cases genetic lethality (by removing the entire gene) may be hard to translate into pharmaceutical effectiveness as drugs can hardly achieve perfect or complete inhibition. In the case of PyDMT1, even half inhibition will effectively suppress its growth and pathogenesis. This level of reduction is more attainable in drug development or realistic in clinical applications.

The complete rescue of our PyDMT1 hypomorphic mutants by iron indicates that the physiological function of PyDMT1 is providing iron for the use of the parasites. Interestingly, zinc could also confer partial rescue. We interpret this as zinc could partially substitute iron’s function in some biological processes within the parasites, instead as that PyDMT1 is physiologically indispensable for both iron and zinc transportation. Notably, a previous study has shown *PbZIPCO* mutant could be rescued by either iron or zinc supplements. However, in neither case, the rescue was incomplete, suggesting ZIPCO functionally acts as both an iron and zinc transporter at the liver stage [5].

Iron addition to the animal resulted in no rescue to the hypomorphic PyDMT1 parasites. It turned out that in vivo external iron dextran may not be effective in increasing the iron level in RBCs and reticulocytes, either due to poor iron uptake by these cells or an inefficient iron source. We did also try FeSO_4_ injection, but this was lethal to the animal. Ferritin knockout, on the other hand, readily elevated the LIP of reticulocytes, leading to a complete rescue. We were somewhat surprised to find that systemic (whole body) removal of ferritin inhibited the growth of the wild-type parasite. One possibility for this host repression effect is that when ferritin was degraded, the stored iron would be released, generating much ROS stress across the system [33, 34]. The ROS pressure from the host would then enforce a potent inhibition on the growth of the parasites.

## Conclusions

*Plasmodium* DMT1 homolog is critical for the iron uptake of the parasites. Even partial loss of function of PyDMT1 appears damaging, suggesting intracellular iron is near the threshold within the normal parasite even though it dwells in a heme-rich environment. This also suggests that there are no redundant pathways for the malarial parasite to acquire iron, and depleting iron, or more specifically, targeting DMT1, might be an effective means of repressing the parasites.

## Methods

### PyDMT1 expression in S. cerevisiae

*△smf3* yeast was generated by transforming a linear DNA fragment containing flanking homologous arms and the Leu2 selectable marker. The *FET3-LacZ* reporter plasmid was constructed by replacing the *CTR3* promoter region of *CTR3-LacZ* plasmid with that of *FET3* (− 300 bp–6 bp). A codon-optimized version of *Pydmt1* ORF was used for yeast expression. The *Pydmt1* expression plasmid was constructed by subcloning the codon-optimized *Pydmt1* fragment into the expression vector pYES2-ADH (KpnI/EcoRI), containing Leu2 as the selectable marker. The N-terminal truncated PyDMT1 expression plasmid was made by PCR amplification of *Pydmt1* and subcloned into the pYES2-ADH. PyDMT1 and the N-terminal truncated PyDMT1 expression plasmids as well as the pYES2-ADH vector were transformed into *△smf3* yeast.

### LacZ activity measurement

LacZ activity of yeast was measured by the kit from Beyotime Biotechnology, following the manufacturer’s instructions.

### Preparation of vacuolar membrane vesicles from yeast

Yeast vacuolar vesicles were isolated from *△smf3* yeast and *△smf3* expressing PyDMT1 strains in parallel according to a previously described protocol [6]. Iron was assayed with ICP-MS (analyzed by ZhongKeBaiCe Co. China).

### Animals

All animal procedures and experiments were performed following the guidelines of the Laboratory Animal Research Center of Tsinghua University. Protocols (17-ZB1) for mouse experiments were approved by the Institutional Animal Care and Use Committee of Tsinghua University. BALB/c mice were purchased from a company (Charles River, China). *Fth*^*fl/fl*^ and *R26*^*CreERT2*^ mice were purchased from Jackson Lab.

### P. yoelii transfection

Transfection experiments were performed with *P. yoelii* 17X strain according to the described protocol [35]. The CRISPR-Cas9 vector pYCm for *Pydmt1* knocking out is a gift from Jing Yuan lab of Xiaman University. The *Pydmt1* knockdown vector was constructed for a single crossover homologous recombination, as previously described [21]. Primer sequences used to amplify the upstream of *Pydmt1* are given in Additional File 1: Table S2. The final knockdown construct was digested with NheI to release the fragment for transfection. The pyrimethamine-resistant parasite population containing the correct genomic integration of the *Pydmt1* knockdown construct was cloned by injecting on average one parasite per mouse (BALB/c male mice, 6–8 weeks of age).

### Genotype analysis of P. yoelii transfectants

PCR analysis performed on the genomic DNA isolated from transgenic *P. yoelii* was used to inspect if the transfection construct was integrated into the correct loci in pyrimethamine-resistant parasites. Primers are listed in Additional File 1: Table S3.

### qRT-PCR

When parasitemia reached about 10%, infected mice were sacrificed by euthanasia, and the whole blood was collected from the animals by cardiac puncture. RBCs were lysed using 1% saponin in phosphate buffered saline (PBS). The parasites were then washed three times with PBS and resuspended in 1 mL TRIzol for 5 min at room temperature followed by 200 μL chloroform treatment. After centrifugation, the aqueous phase was transferred to a new Eppendorf tube containing 400 μL isopropanol to precipitate RNA. cDNA was reverse-transcribed from total RNA with the Transcriptor First strand cDNA Synthesis Kit (Transgene, China). Quantitative real-time PCR was performed using 1 μg cDNA in duplicate under the following conditions: 95 °C/10 min, followed by 40 cycles of 95 °C/15 s (denaturation), 55 °C/30 s (annealing) and 72 °C/30 s (elongation). PCR primers are listed in Additional File 1:Table S4.

### Western blotting

Total proteins extracted from parasite pellets were separated on 4 to 15% SDS–polyacrylamide gels and transferred to polyvinylidene difluoride (PVDF) membranes (Millipore). The blot was incubated with blocking buffer (PBST with 5% milk powder) at room temperature for 1 h and then incubated at 4 °C overnight with anti-HA (rabbit; 1:1000; Cell Signaling) and anti-Bip from Yuan Jing lab (rabbit; 1:1000). Antibody to Bip was used as a control. Horseradish peroxidase-conjugated goat anti-rabbit or anti-mouse antibodies (Sigma) were incubated with PVDF membranes for 2 h at room temperature before three washes with blocking and enhanced chemiluminescence (ECL) detection.

### Fluorescence analysis of recombinant parasites

The parasites were washed twice with PBS and were fixed with 4% paraformaldehyde on polyL-lysine-coated glass slides for 30 min. After 3 washes in PBS, the parasites were treated with 0.1% Triton X-100 in PBS, blocked with 3% BSA in PBS, and stained with the primary antibody (mouse anti-Flag [1:1000; Cell Signal], rabbit anti-HA [1:500; Cell Signal] at 4 °C overnight. After 3 washes with blocking buffer, the samples were incubated with goat anti-mouse or anti-rabbit antibody labeled with Alexa Fluor 488 (1:2000; Invitrogen) at room temperature for 1 h. Cells were stained with Hoechst 33342 to visualize nuclei. All images were captured and processed using identical settings in the Olympus FV3000 laser scanning confocal microscope with a 100/1.49-numerical-aperture (NA) oil objective. Similar results were obtained in at least three independent experiments.

### In vitro maturation assays

Rodent malaria parasites at blood stages can only be maintained in vitro for one developmental cycle, allowing parasites to mature from rings/young trophozoites into segmented schizonts in culture medium supplemented with iron or zinc [36]. Parasites were collected from the tails of infected mice at 0.5–2% parasitemia, washed in warmed incomplete RPMI1640 and added to 96-well plates with complete culture medium (incomplete RPMI1640 supplemented with 25 mM HEPES, 2 g NaHCO_3_, 50 μg gentamicin and 20%FBS) at 1% hematocrit(final volume 100μL). Plates were incubated at 37 °C in an atmosphere of 5% O_2_, 5% CO_2_ and 90% N_2_ for 18 h. Mature schizonts were visualized by microscopy after Giemsa staining and quantified manually.

### Determination of the LIP in iRBCs

The LIPs of RBCs infected with the control and *Pydmt1* mutant parasites were determined by flow cytometry using the Calcein-AM fluorescent iron probe [37]. Parasite-infected BALB/c male mice (6–8 weeks of age) with 5–10% parasitemia were bled and the RBCs were incubated 1 h with 1 μM Calcein-AM and 5 μM Hoechst 33342 in the presence and absence of 100 μM DIP. Following two washes in PBS, stained cells were analyzed on a FACS Calibur (Beckman Coulter, Inc). The amount of labile iron was estimated for each sample relative to that with DIP (△MFI). Images of iRBCs infected with *PyDMT1-HA* or *Pydmt1*-A1 were collected with an Olympus confocal microscope (FV3000). The mean fluorescence intensity of Calcein-AM in the region of parasite was calculated by the software of FV31S-SW supplied by Olympus.

### Inducible gene deletion of Fth1

Inducible deletion of the *Fth*^*fl/fl*^ allele in R26^CreERT2^ *Fth*^*△/△*^ mice was achieved at 6–8 weeks of age by tamoxifen (MCE. China) intraperitoneal injection (100 mg/kg BW in 100 μL peanut oil, once a day for 5 consecutive days). Weight was monitored daily. Control *Fth*^*fl/fl*^ maintained under specific pathogen free conditions also received the same tamoxifen treatment.

### ELISA to measure ferritin

ELISA kits (Abcam) were used to measure ferritin levels according to the manufacturer’s protocols.

### Ferrozine-based iron content assay

One hundred microliters blood was collected from facial vein to heparin-coated tube and then was centrifugated to separate RBCs and plasma. About 50 µL RBCs were homogenized in 1% NP-40 buffer and then samples were centrifuged to remove insoluble pellet. Protein contents of plasma and RBC lysates were quantified by the BCA Protein Assay Kit. Iron was released by mixing aliquots (50 µL RBCs lysates, for plasma, 10 µL plus 40 µL saline solution) of the lysates with 11 µL of concentrated hydrochloric acid (HCl) and incubated for 20 min at 95 °C (free iron detection omits this step). After centrifuging at 15,000 rpm, 45 µL of the supernatant was collected and then mixed with 36 µL of the iron release buffer (37.5 mM ascorbate, 5 mM ferrozine). Finally, 36 µL of saturated ammonium acetate was added into each tube and the absorbance at 562 nm was measured using a plate reader (Thermo Scientific Multiskan GO).

### Drug sensitivity in vivo

3 × 10^^^6 iRBC of *PyDMT1-HA* and 1 × 10^^^6 iRBC of *Pydmt1*-A1 were respectively injected into BALB/c mouse (day 0). Parasitemia of *PyDMT1-HA* and *Pydmt1*-A1 reached to around 10% at the same time at day 3. From day 3 onward, mice were treated with curative doses of artemisinin (7.5 mg/kg body weight, i.p.), artesunate (8 mg/kg body weight, i.p.), chloroquine (6 mg/kg body weight, i.p.). Subsequent parasitemia values were determined daily by blood smear of peripheral blood. Inhibition rates were calculated as the following formula, wherein “A” indicates parasitemia before drug administration and “B” represents parasitemia after drug treatment.

$$Inhibition rate = \frac{A-B}{B} \times 100\%$$

### Supplementary Information

**Additional file 1:** **Figure S1.** Strategy for PyDMT1-HA (a) and PyCRT-FLAG (c) constructions. Briefly, integration of a gene fusion construct is generated after a Cas9-mediated cut in the genome, followed by homologous recombination and drug selection. Genotyping of PyDMT1-HA (b) and PyCRT-FLAG (d) transgenic pool and clonal lines by PCR. Primers P3/P4, detection of construct integration at the 5’-end; primers P5/P6, construct integration detection used at the 3’-end. Primers P7/P8, detection of integration at the 5’-end; primers P9/P10, integration detection at the 3’-end. The left is a pool, and the right is clones after negative selection. *Pydmt1* transcription level (e) and parasitemia (f) of PyDMT1-HA transgenic clones B3 and B5. The localization of PyDMT1-HA with PyPMV-FLAG (g) and PyRab6-FLAG (h) in the blood stage. Strategy for knocking out SMF3 constructions in yeast (i). **Figure S2.** Genotyping of the PyDMT1 knockdown (hypomorph) transgenic parasites by PCR (a). The mRNA level of flanking gene PY_17X1241700 in *Pydmt1*-A1 and *Pydmt1*-C2 parasites (c). The HA antibody specifically detects PyDMT1-HA fusion protein (d). Gating strategy applied to select iRBC of PyDMT1-HA and clone A1 parasites after DIP treatments. Parasite-infected red blood cells are Hoechst 33342-positive (e). **Figure S3.** Iron contents of the plasma and red blood cell in the mice after 100 mg/kg iron dextran injection (a). Parasitemia of C57BL/6 mice infected with control and *Pydmt*1-A1 parasites after iron-dextran injection (b). **Table S1** Guide RNAs designed for *Pydmt1* knock out. **Table S2** PyDMT1 knockdown primers. **Table S3** Primers for detection of transgene parasites. **Table S4** Primers for qPCR.

## Data Availability

All data generated or analyzed during this study are included in this published article and its supplementary information files.

## Abbreviations

DMT1: Divalent metal transporter 1
HO: Heme oxygenase
ZIP: Zinc-regulated, iron-regulated transporter-like proteins
ZIPCO: ZIP domain-containing protein
VIT: Vacuolar iron transporter
CCC1: Cross-complementer of CSG1 protein 1
CRT: Chloroquine resistance transporter
HA: Hemagglutinin
RBC: Red blood cell
LIP: Iron labile pool
DIP: 2,2'-Dipyridyl
FVRT1: Food vacuole resident transporter 1
ROS: Reactive oxygen species
PBS: Phosphate buffered saline

## References

[1] Rosenthal PJ, Meshnick SR (1996). Hemoglobin catabolism and iron utilization by malaria parasites. Mol Biochem Parasitol.

[2] Scholl PF, Tripathi AK, Sullivan DJ, Sullivan DJ, Krishna S (2005). Bioavailable iron and heme metabolism in Plasmodium falciparum. Malaria: drugs, disease and post-genomic biology.

[3] Wilks A (2002). Heme oxygenase: evolution, structure, and mechanism. Antioxid Redox Signal.

[4] Sigala PA, Crowley JR, Hsieh S, Henderson JP, Goldberg DE (2012). Direct tests of enzymatic heme degradation by the malaria parasite Plasmodium falciparum. J Biol Chem.

[5] Sahu T, Boisson B, Lacroix C, Bischoff E, Richier Q, Formaglio P, Thiberge S, Dobrescu I, Menard R, Baldacci P (2014). ZIPCO, a putative metal ion transporter, is crucial for Plasmodium liver-stage development. EMBO Mol Med.

[6] Slavic K, Krishna S, Lahree A, Bouyer G, Hanson KK, Vera I, Pittman JK, Staines HM, Mota MM (2016). A vacuolar iron-transporter homologue acts as a detoxifier in Plasmodium. Nature Commun.

[7] Bakouh N, Bellanca S, Nyboer B, Moliner Cubel S, Karim Z, Sanchez CP, Stein WD, Planelles G, Lanzer M (2017). Iron is a substrate of the Plasmodium falciparum chloroquine resistance transporter PfCRT in Xenopus oocytes. J Biol Chem.

[8] Slater AF (1993). Chloroquine: mechanism of drug action and resistance in Plasmodium falciparum. Pharmacol Ther.

[9] Spottiswoode N, Duffy PE, Drakesmith H (2014). Iron, anemia and hepcidin in malaria. Front Pharmacol.

[10] Gwamaka M, Kurtis JD, Sorensen BE, Holte S, Morrison R, Mutabingwa TK, Fried M, Duffy PE (2012). Iron deficiency protects against severe Plasmodium falciparum malaria and death in young children. Clin Infect Dis.

[11] Sazawal S, Black RE, Ramsan M, Chwaya HM, Stoltzfus RJ, Dutta A, Dhingra U, Kabole I, Deb S, Othman MK (2006). Effects of routine prophylactic supplementation with iron and folic acid on admission to hospital and mortality in preschool children in a high malaria transmission setting: community-based, randomised, placebo-controlled trial. Lancet.

[12] Portugal S, Carret C, Recker M, Armitage AE, Goncalves LA, Epiphanio S, Sullivan D, Roy C, Newbold CI, Drakesmith H (2011). Host-mediated regulation of superinfection in malaria. Nat Med.

[13] Zhang DL, Wu J, Shah BN, Greutelaers KC, Ghosh MC, Ollivierre H, Su XZ, Thuma PE, Bedu-Addo G, Mockenhaupt FP (2018). Erythrocytic ferroportin reduces intracellular iron accumulation, hemolysis, and malaria risk. Science.

[14] Mims MP, Prchal JT (2005). Divalent metal transporter 1. Hematology.

[15] Andrews NC (1999). The iron transporter DMT1. Int J Biochem Cell Biol.

[16] Yanatori I, Kishi F (2019). DMT1 and iron transport. Free Radical Biol Med.

[17] Zhang C, Xiao B, Jiang Y, Zhao Y, Li Z, Gao H, Ling Y, Wei J, Li S, Lu M (2014). Efficient editing of malaria parasite genome using the CRISPR/Cas9 system. mBio.

[18] Zhang C, Gao H, Yang Z, Jiang Y, Li Z, Wang X, Xiao B, Su XZ, Cui H, Yuan J (2017). CRISPR/Cas9 mediated sequential editing of genes critical for ookinete motility in Plasmodium yoelii. Mol Biochem Parasitol.

[19] Sanchez CP, Stein WD, Lanzer M (2007). Is PfCRT a channel or a carrier? Two competing models explaining chloroquine resistance in Plasmodium falciparum. Trends Parasitol.

[20] Portnoy ME, Liu XF, Culotta VC (2000). Saccharomyces cerevisiae expresses three functionally distinct homologues of the nramp family of metal transporters. Mol Cell Biol.

[21] Nair SC, Xu R, Pattaradilokrat S, Wu J, Qi Y, Zilversmit M, Ganesan S, Nagarajan V, Eastman RT, Orandle MS (2017). A Plasmodium yoelii HECT-like E3 ubiquitin ligase regulates parasite growth and virulence. Nat Commun.

[22] Clark M, Fisher NC, Kasthuri R, Cerami Hand C (2013). Parasite maturation and host serum iron influence the labile iron pool of erythrocyte stage Plasmodium falciparum. Br J Haematol.

[23] Knovich MA, Storey JA, Coffman LG, Torti SV, Torti FM (2009). Ferritin for the clinician. Blood Rev.

[24] Arosio P, Elia L, Poli M (2017). Ferritin, cellular iron storage and regulation. IUBMB Life.

[25] Ferreira C, Bucchini D, Martin ME, Levi S, Arosio P, Grandchamp B, Beaumont C (2000). Early embryonic lethality of H ferritin gene deletion in mice. J Biol Chem.

[26] Blankenhaus B, Braza F, Martins R, Bastos-Amador P, Gonzalez-Garcia I, Carlos AR, Mahu I, Faisca P, Nunes JM, Ventura P (2019). Ferritin regulates organismal energy balance and thermogenesis. Mol Metab.

[27] Ney PA (2011). Normal and disordered reticulocyte maturation. Curr Opin Hematol.

[28] Ogawa C, Tsuchiya K, Maeda K (2020). Reticulocyte hemoglobin content. Clin Chim Acta.

[29] Stevens-Hernandez CJ, Bruce LJ (2022). Reticulocyte Maturation. Membranes (Basel).

[30] Everds EN, Fox JG, Davisson MT, Quimby FW, Barthold SW, Newcomer CE, Smith AL (2007). Chapter 5 - Hematology of the laboratory mouse. The mouse in biomedical research (Second Edition).

[31] Li J, Zhou B (2010). Biological actions of artemisinin: insights from medicinal chemistry studies. Molecules.

[32] Wichers Jan S, Mesén-Ramírez P, Fuchs G, Yu-Strzelczyk J, Stäcker J, von Thien H, Alder A, Henshall I, Liffner B, Nagel G (2022). PMRT1, a Plasmodium-specific parasite plasma membrane transporter, is essential for asexual and sexual blood stage development. mBio.

[33] Winterbourn CC (1995). Toxicity of iron and hydrogen peroxide: the Fenton reaction. Toxicol Lett.

[34] Muckenthaler MU, Rivella S, Hentze MW, Galy B (2017). A red carpet for iron metabolism. Cell.

[35] Janse CJ, Ramesar J, Waters AP (2006). High-efficiency transfection and drug selection of genetically transformed blood stages of the rodent malaria parasite Plasmodium berghei. Nat Protoc.

[36] Mancio-Silva L, Slavic K, Grilo Ruivo MT, Grosso AR, Modrzynska KK, Vera IM, Sales-Dias J, Gomes AR, MacPherson CR, Crozet P (2017). Nutrient sensing modulates malaria parasite virulence. Nature.

[37] Darbari D, Loyevsky M, Gordeuk V, Kark JA, Castro O, Rana S, Apprey V, Kurantsin-Mills J (2003). Fluorescence measurements of the labile iron pool of sidde erytluocytes. Blood.

