# Supplementary material for: Plasmodium yoelii iron transporter PyDMT1 interacts with host ferritin and is required in full activity for malarial pathogenesis
Source: BMC Biol. 2023 Dec 5;21:279. doi: 10.1186/s12915-023-01776-y (PMC10696721; doi:10.1186/s12915-023-01776-y)
Supplement: Supplementary file 1 — Additional file 1: Figure S1. Strategy for PyDMT1-HA (a) and PyCRT-FLAG (c) constructions. Briefly, integration of a gene fusion construct is generated after a Cas9-mediated cut in the genome, followed by homologous recombination and drug selection. Genotyping of PyDMT1-HA (b) and PyCRT-FLAG (d) transgenic pool and clonal lines by PCR. Primers P3/P4, detection of construct integration at the 5’-end; primers P5/P6, construct integration detection used at the 3’-end. Primers P7/P8, detection of integration at the 5’-end; primers P9/P10, integration detection at the 3’-end. The left is a pool, and the right is clones after negative selection. Pydmt1 transcription level (e) and parasitemia (f) of PyDMT1-HA transgenic clones B3 and B5. The localization of PyDMT1-HA with PyPMV-FLAG (g) and PyRab6-FLAG (h) in the blood stage. Strategy for knocking out SMF3 constructions in yeast (i). Figure S2. Genotyping of the PyDMT1 knockdown (hypomorph) transgenic parasites by PCR (a). The mRNA level of flanking gene PY_17X1241700 in Pydmt1-A1 and Pydmt1-C2 parasites (c). The HA antibody specifically detects PyDMT1-HA fusion protein (d). Gating strategy applied to select iRBC of PyDMT1-HA and clone A1 parasites after DIP treatments. Parasite-infected red blood cells are Hoechst 33342-positive (e). Figure S3. Iron contents of the plasma and red blood cell in the mice after 100 mg/kg iron dextran injection (a). Parasitemia of C57BL/6 mice infected with control and Pydmt1-A1 parasites after iron-dextran injection (b). Table S1 Guide RNAs designed for Pydmt1 knock out. Table S2 PyDMT1 knockdown primers. Table S3 Primers for detection of transgene parasites. Table S4 Primers for qPCR. [file 12915_2023_1776_MOESM1_ESM.docx]

**Supplementary Information**

**Fig S1**


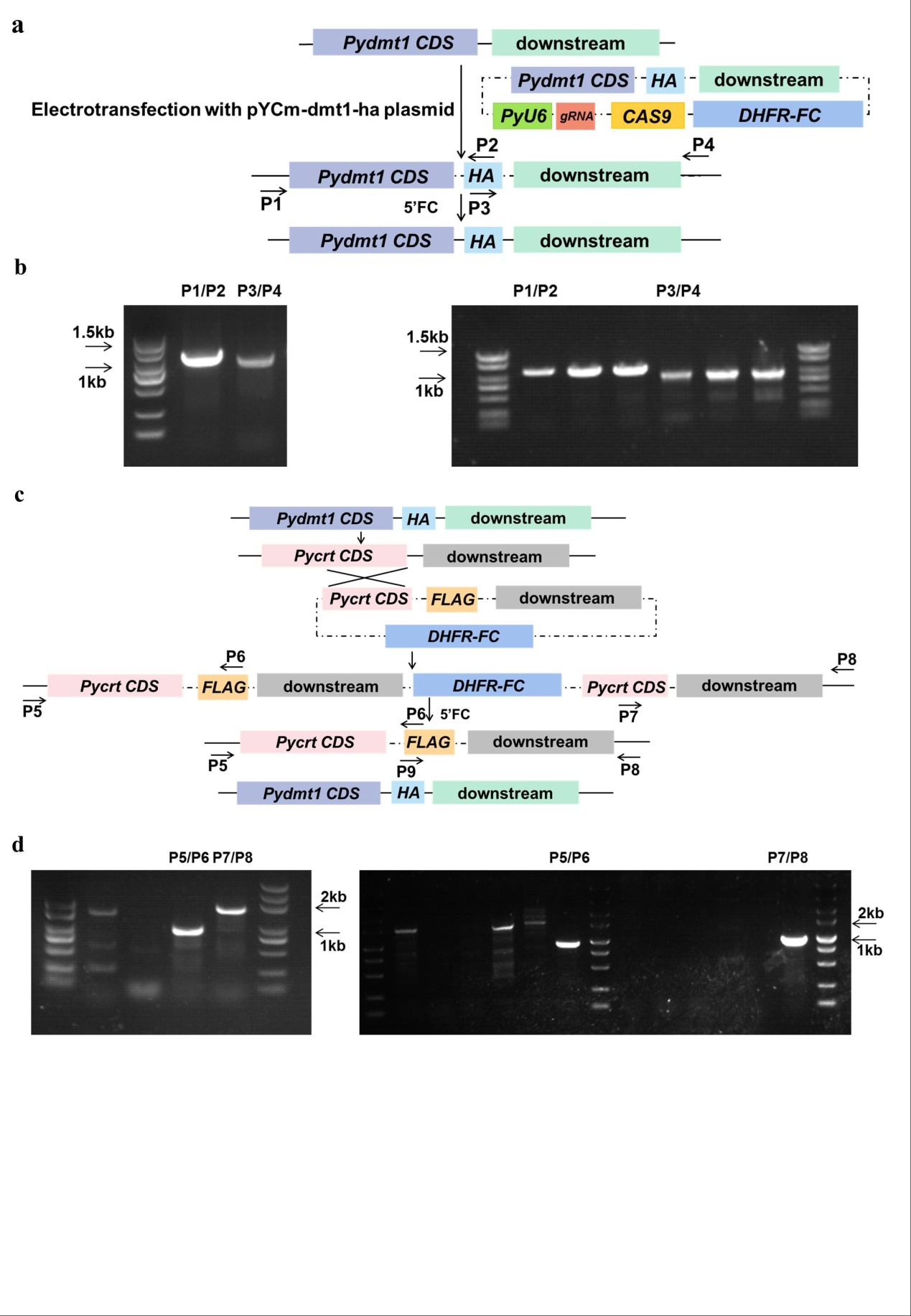


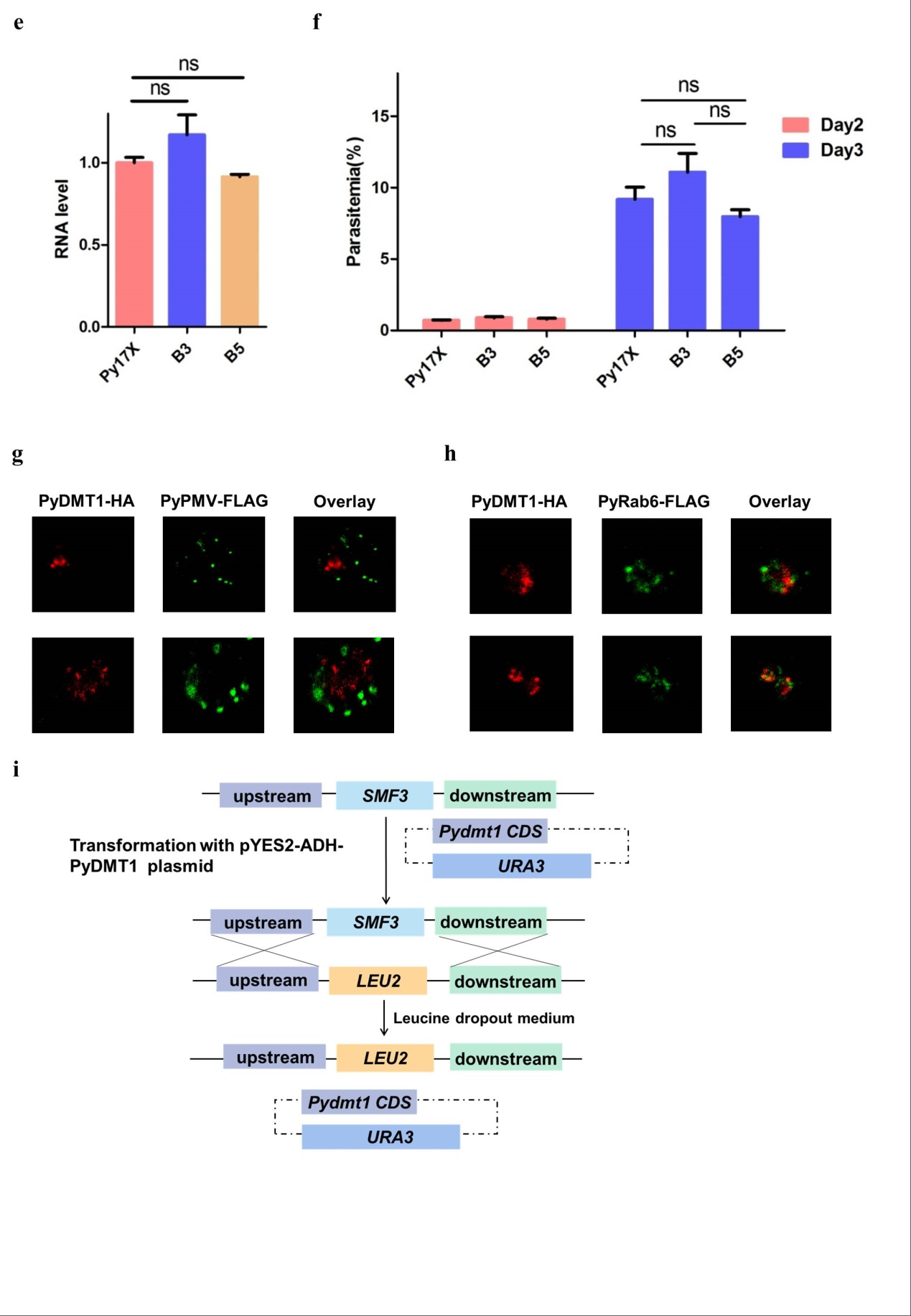


FigS1

(a) Strategy for PyDMT1-HA constructions in *P. yoelii*. Briefly, integration of a gene fusion construct is generated after a Cas9-mediated cut in the genome, followed by homologous recombination and drug selection.(b) Genotyping of PyDMT1-HA transgenic pool and clonal lines by PCR. Primers P3/P4, detection of construct integration at the 5’-end; primers P5/P6, construct integration detection used at the 3’-end.(c) Strategy for PyCRT-FLAG constructions in PyDMT1-HA parasites. Integration of *FLAG* is generated by a single crossover, followed by negative selection of 5’-FC(Fluorocytosine).(d) Genotyping of PyCRT-FLAG transgenic lines by PCR. Primers P7/P8, detection of integration at the 5’-end; primers P9/P10, integration detection at the 3’-end. The left is a pool, and the right is clones after negative selection.(e) RNA Levels of PyDMT1-HA transgenic clones B3 and B5, normalized with tubulin (N=3，one way ANOVA and Tukey’s multiple comparison test. n.s. P>0.05).(f) Parasitemia of BALB/c mice infected with wild type *P. yoelii*-17X, B3 and B5 strains (N=5，one way ANOVA and Tukey’s multiple comparison test. n.s. P>0.05).(g) Indirect immunofluorescence assays of PyDMT1-HA and PyRab6-FLAG in the blood stage. The endogenous PyDMT1 was fused to HA. HA and the Glogi marker PyRab6-FLAG were imaged. Shown are the HA channel(red), the FLAG channel (green), and a merge of both signals.(h) Indirect immunofluorescence assays of PyDMT1-HA and PyPMV-FLAG in the blood stage. The PyDMT1-HA and the endoplasmic reticulum marker *PyRab6-FLAG* were imaged. Shown are the HA channel(red), the FLAG channel(green), and a merge of both signals.(i) Strategy for knocking-out *SMF3* and complementation with PyDMT1 in the yeast*.*

**Fig S2**


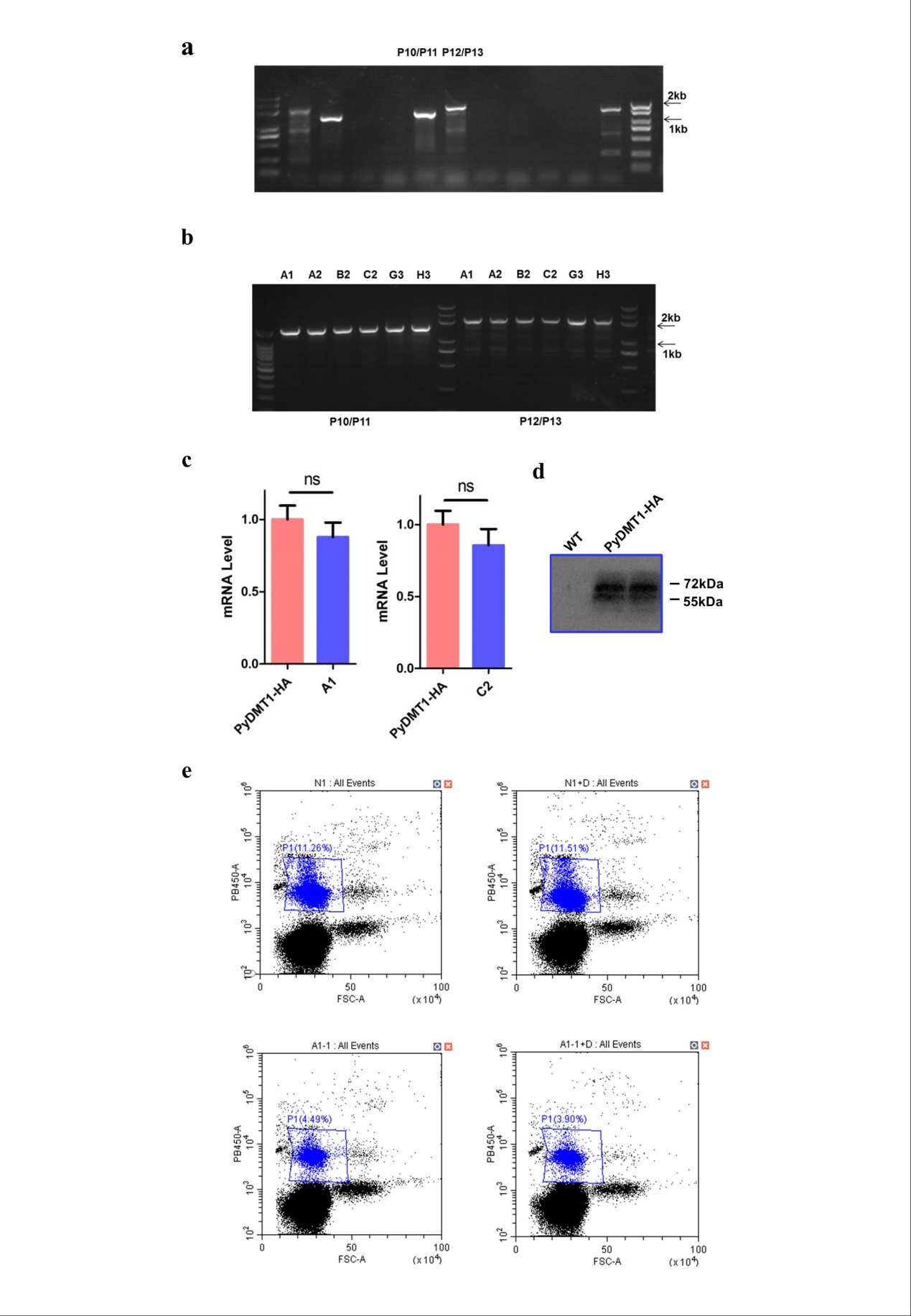


FigS2

(a, b) Genotyping of the PyDMT1 knockdown(hypomorph) transgenic parasites by PCR. (b) is transgenic clonal lines by PCR. Primers P12/P13, detection of knockdown construct integration at the 5’-end; primers P13/P14, knockdown construct integration detection at the 3’-end.(c) The mRNA level of flanking gene PY_17X1241700 in *Pydmt1-A1* and *Pydmt1-C2* parasites, normalized by tubulin(t-test, n.s. no significance).(d) The HA antibody specifically detects PyDMT1-HA fusion protein. Lane 1(WT) was protein lysate of *P. yoelii 17X* parasites, and lane 2 lysate of PyDMT1-HA parasites. Comparable protein concentrations were loaded each lane. In the WT lane, no bands were detected, suggesting the bands in the PyDMT1-HA lanes were all related to the PyDMT1-HA fusion.(e) Gating strategy applied to select iRBC of PyDMT1-HA and clone A1 parasites after DIP treatments. Parasite-infected red blood cells are Hoechst 33342-positive(blue square).

**Fig S3**


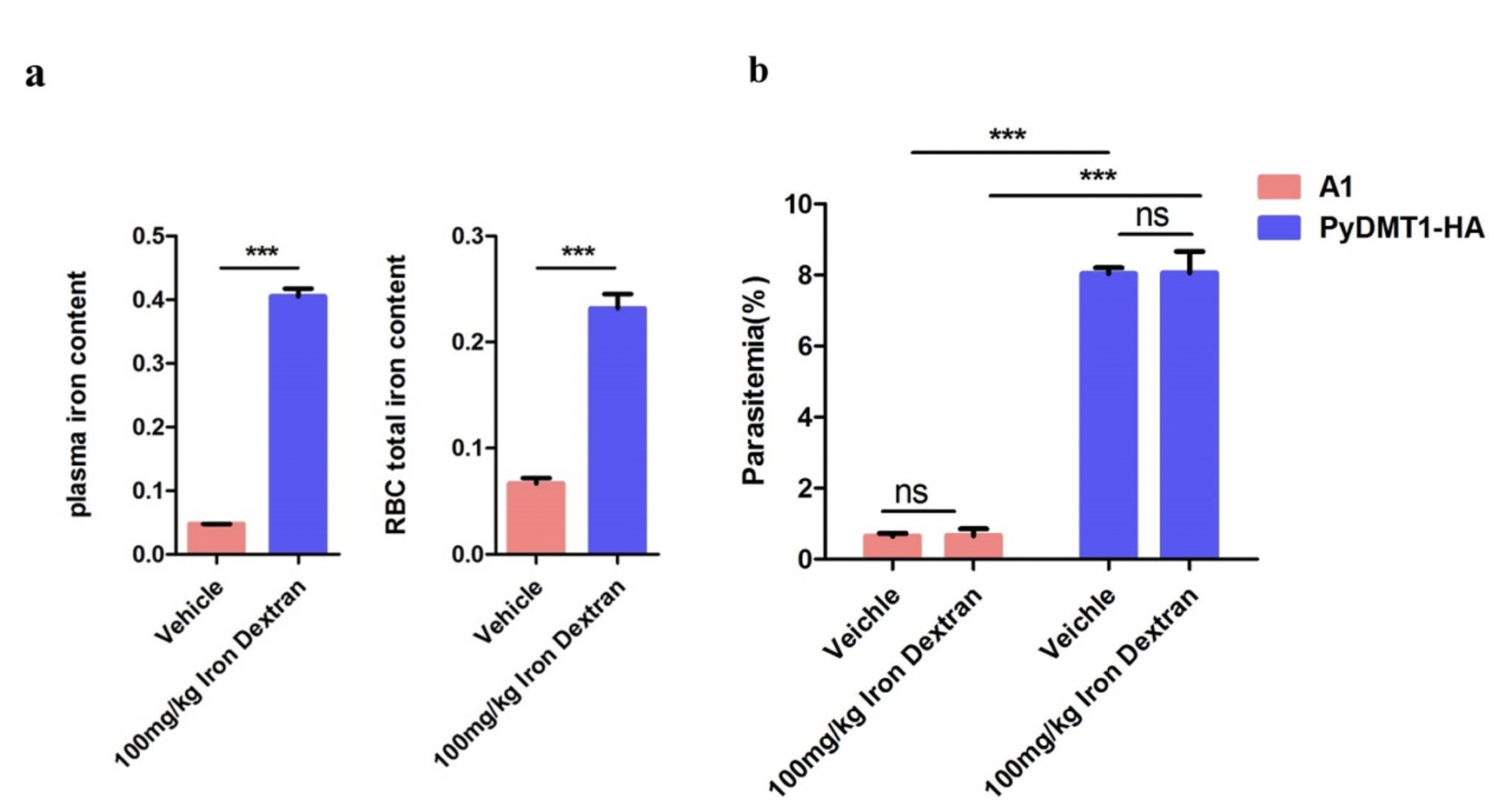


# FigS3

# (a) Iron contents of the plasma and red blood cell in the mice after 100 mg/kg iron dextran injection(N=3, t-test,***P<0.001).(b) Parasitemia of C57BL/6 mice infected with control and *Pydmt*1-A1 parasites after iron-dextran injection(N=5, one way ANOVA and Tukey’s multiple comparison test. n.s. P>0.05, ***P<0.001).

# TableS1

Knock out guide RNAs

| **guide RNA** | **Sequence** |
| --- | --- |
| gRNA1 | TGTGTGCAAATAGCTATTTG |
| gRNA2 | CAATCAACTGCATATTGTGA |
| gRNA3 | TTGGGAACAATCGAATCGGG |
| gRNA4 | GCAATAGCAACAATCCATCC |
| gRNA5 | GGGCTCCCCAAATAGCTATT |
| gRNA6 | TAAGTTGGGAACAATCGAAT |
| gRNA7 | AAGTTGGGAACAATCGAATC |
| gRNA8 | AATAGCTATTTGGACACATA |
| gRNA9 | TTAGACCCTGGTAATATATG |

TableS2

PyDMT1 knockdown primers

| Primer name | Primer sequence |
| --- | --- |
| PL0019-1 | cctcttcgctattacgccagCGTGATTATATATTTATTGTTCAGACTTG |
| PL0019-400-2 | ctggttgatcttgccagtagtcGTGACTAATTTGTAGTATGCGTTC |
| PL0019-600-2 | ctggttgatcttgccagtagtcGGTTCGCTTAAACTGAC |
| PL0019-800-2 | ctggttgatcttgccagtagtcATGATTTTAGTCTGTATATATATATTTTG |
| PL0019-1200-2 | cctcttcgctattacgccagGTCATTCTTTTATTATTATTATATC |
| PL0019-1600-2 | cctcttcgctattacgccagCCAAAATTCGTTGAATTATGATAAT |
| PL0019-2000-2 | cctcttcgctattacgccagGCATTATTAATTTTTATAGTGTTCATTCT |

TableS3

Primers for detection of transgene parasites

| Primer name | Primer sequence |
| --- | --- |
| P1 | TTGATAGAAGCATTTGCATAC |
| P2 | CTAGAAGCGTAATCTGGAAC |
| P3 | CCATATGACGTTCCAGATTA |
| P4 | TACTGTAGGCTTATTTGT |
| P5 | GAACTGTGGTGTTGGC |
| P6 | CTTGTAGTCTCCGTCG |
| P7 | CTGGTTGATCTTGCCAGTAGTC |
| P8 | GAAAACATAGCCATATTCATC |
| P9 | CGACGGAGACTACAAG |
| P10 | GTTGATATAAGTTACACTCGC |
| P11 | CTGGTTGATCTTGCCAGTAGTC |
| P12 | CCTCTTCGCTATTACGCCAG |
| P13 | TCTTCTCATCGAGTTGTCTTG |

TableS4

Primers for qPCR

| Primer name | Primer sequence |
| --- | --- |
| RT-NRAMP-1616-F | GTAGTGGAAATAATGCAAGC |
| RT-NRAMP-1872-R | CTCCTTTAATAGCAAACTTTCC |
| RT-1241700-F | GGATAGTGCAACCACgtttga |
| RT-1241700-R | CACATACCACATAATTTATAGGTAC |
| RT-TUBLIN-F | cATGGAATAGATCCAagcggt |
| RT-TUBLIN-R | tCCTGCTCCAGTTTGTCC |
| RT-mouse actin-F | GCCACTGCCGCATCCTCTTC |
| RT-mouse actin-R | AGCCTCAGGGCATCGGAACC |
| RT-mouse Fpn-F | ATGGGAACTGTGGCCTTCAC |
| RT-mouse Fpn-R | TCCAGGCATGAATACGGAGA |
| RT-mouse Ferritin-F | CCATCAACCGCCAGATCAAC |
| RT-mouse Ferritin-R | GCCACATCATCTCGGTCAAA |
| RT-mouse Tfr1-F | GTTTTTGTGAGGATGCAGACTATCC |
| RT-mouse Tfr1-R | GCTGAGGAACTTTCTGAGTCAATG |
| RT-mouse DMT1-F | GCAGTGGTTAGCGTGGCTTATT |
| RT-mouse DMT1-R | AGACAGACCCAATGCAATCAAA |
